# Supplementary material for: Anti-Inflammatory Activity of Bryophytes Extracts in LPS-Stimulated RAW264.7 Murine Macrophages
Source: Molecules. 2022 Mar 17;27(6):1940. doi: 10.3390/molecules27061940 (PMC8953629; doi:10.3390/molecules27061940)
Supplement: Supplementary file 1 [file molecules-27-01940-s001.zip › molecules-1616276-supplementary.pdf]

Supplementary Material

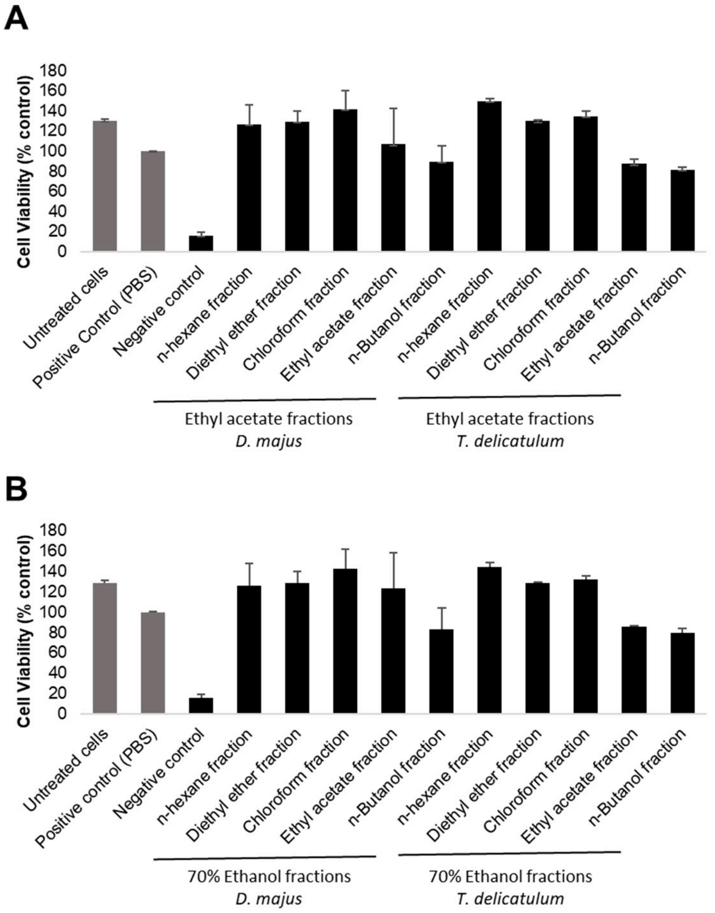

Figure S1. Effect of 70% ethanol and ethyl acetate derived fractions on the viability of RAW 264.7 cells determined by MTT assay. **A**) Ethyl acetate derived fractions from *D. majus* and *T. delicatulum* and **B**) 70% ethanol derived fractions from *D. majus* and *T. delicatulum.* Cells were treated with 10 µg/mL of fractions for 24 h. Data represent mean ± standard deviation (n= 2-3) and values are normalized on control (PBS). Negative control (10% DMSO).

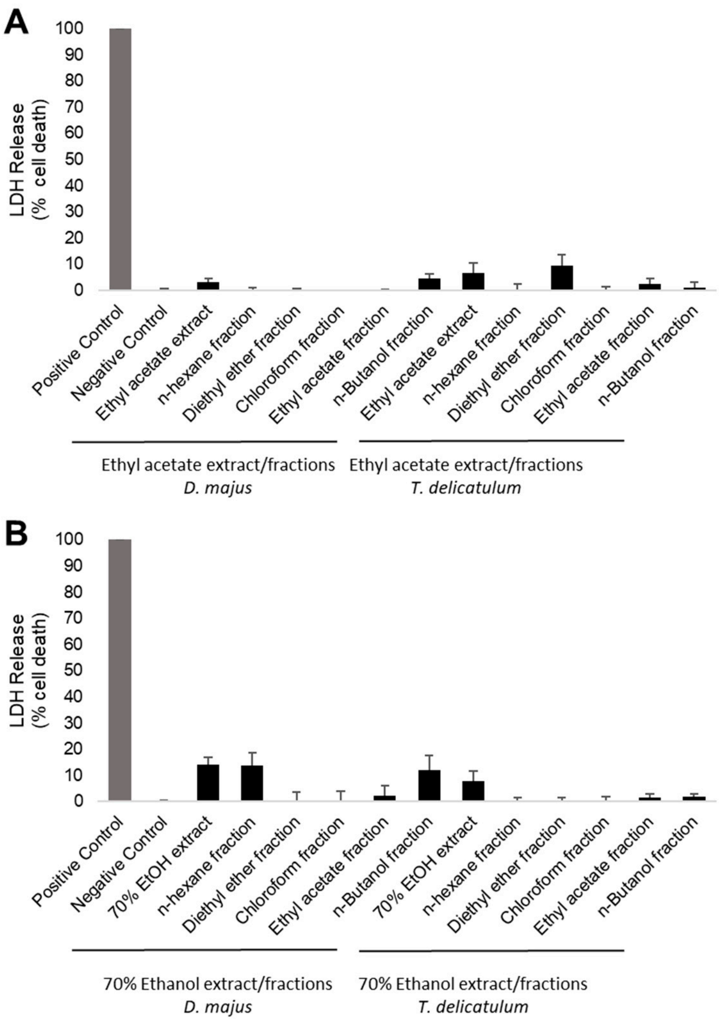

Figure S2. Cell death assessed by lactate dehydrogenase (LDH) release assay after treatment with bioactive whole extracts and derived fractions. **A**) Ethyl acetate extracts and derived fractions of *D. majus* and *T. delicatulum*. **B**) 70% ethanol extracts and derived fractions of *D. majus* and *T. delicatulum*. Cells were treated with 10 µg/mL of extracts/fractions for 24 h. Data represent mean ± standard deviation (n= 2-3). Positive control (no treated lyzed cells) and negative control (0.1 % DMSO). The tested extracts/fractions revealed significant difference (P-values < 0.05, calculated with ANOVA).

Table S1. List of species screened for anti-inflammatory activity and information on collection area.

Group of bryophytes	Family	Species	Country-collection place	GPS coordinates Latitude	Longitude	Date of collection
**Mosses**						
	Amblystegiaceae	*Warnstorfia fluitans* (Hedw.) Loeske	Iceland	64.14197491	-20.23437432	12 June 2019
	Bartramiaceae	*Philonotis fontana* (Hedw.) Brid.	Iceland	64.14389823	-20.22702004	12 June 2019
	Brachytheciaceae	*Brachythecium rutabulum* (Hedw.) Schimp.	Germany, Black Forest	47.917959	8.075349	28 April 2018
		Cirriphyllum crassinervium (Taylor) Loeske & M. Fleisch.	Denmark, Rude Skov	55.84145	12.47838	24-25 October 2018

		Homalothecium lutescens (Hedw.) H. Rob.	Sweden	55.7168043	13.7214639	10 September 2019
		Homalothecium sericeum (Hedw.) Schimp.	Germany, Black Forest	47.917733	8.075181	28 April 2018
		Kindbergia praelonga (Hedw.) Ochyra	Moss provider (Bryoflor, Paris, France)	-	-	Purchased on 20 February 2019
	Bryaceae	*Bryum pseudotriquetrum* (Hedw.) P. Gaertn., B. Mey. & Scherb.	Germany, Black Forest	47.912221	8.081158	28 April 2018
	Dicranaceae	*Dicranum majus* Turner	Sweden	56.02397	13.13074	27 October 2018

	Dicranum scoparium Hedw.	Denmark, Rude Skov	55.84072	12.47253	24-25 October 2018
Grimmiaceae	*Racomitrium aciculare* (Hedw.) Brid.	Germany, Black Forest	47.910660	8.093621	28 April 2018
	Racomitrium elongatum Ehrh. ex Frisvoll	Sweden	55.7264294	13.70606602	10 September 2019
Hedwigiaceae	*Hedwigia ciliata* (Hedw.) P. Beauv.	Moss provider (Bryoflor, Paris, France)	-		Purchased on 20 February 2019
Hylocomiaceae	*Hylocomium splendens* (Hedw.) Schimp.	Sweden	55.7223757	13.7066895	10 September 2019

	Rhytidiadelphus loreus (Hedw.) Warnst.	Germany, Black Forest	47.911177	8.092482	28 April 2018
	Rhytidiadelphus squarrosus (Hedw.) Warnst.	Denmark, Rude Skov	55.84071	12.47371	24-25 October 2018
	Rhytidiadelphus triquetrus (Hedw.) Warnst.	Iceland	64.32743787	-20.28594921	13 June 2019
	Rhytidiadelphus triquetrus (Hedw.) Warnst.	Sweden	55.721833	13.7021155	10 September 2019
Hypnaceae	*Hypnum cupressiforme* Hedw.	Denmark, Rude Skov	55.84201	12.4748	24-25 October 2018

	Leucobryaceae	*Campylopus introflexus* (Hedw.) Brid.	Moss provider (Bryoflor, Paris, France)	-	-	Purchased on 20 February 2019
	Mniaceae	*Mnium hornum* Hedw.	Denmark, Rude Skov	55.84161	12.47275	24-25 October 2018
		Plagiomnium undulatum (Hedw.) T.J. Kop.	Sweden	55.72548924	13.7051406	10 September 2019
	Neckeraceae	*Thamnobryum alopecurum* (Hedw.) Nieuwl. ex Gangulee	Germany, Black Forest	47.917927	8.074989	28 April 2018
	Plagiotheciaceae	*Plagiothecium undulatum* (Hedw.) Schimp.	Denmark, Rude Skov	55.84915	12.4582	24-25 October 2018

	Polytrichaceae	*Polytrichum formosum* Hedw.	Germany, Black Forest	47.911223	8.092431	28 April 2018
	Pylaisiaceae	*Calliergonella cuspidata* (Hedw.) Loeske	Denmark, Rude Skov	55.84113	12.48188	24-25 October 2018
		Ptilium crista-castrensis (Hedw.) De Not.	Germany, Black Forest	47.911766	8.089162	28 April 2018
	Sphagnaceae	*Sphagnum fimbriatum* Wilson	Denmark, Rude Skov	55.84098	12.47348	24-25 October 2018
		Sphagnum teres (Schimp.) Ångström	Iceland	64.32743787	-20.28594921	13 June 2019

	Thuidiaceae	*Thuidium delicatulum* (Hedw.) Schimp.	Sweden	56.02422	13.12932	27 October 2018
**Liverworts**						
	Plagiochilaceae	*Plagiochila asplenioides* (L.) Dumort.	Germany, Black Forest	47.917927	8.074989	28 April 2018
	Lepidoziaceae	*Bazzania trilobata* (L.) Gray	Germany, Black Forest	47.911223	8.092431	28 April 2018
	Metzgeriaceae	*Metzgeria furcata* (L.) Corda	Denmark, Rude Skov	55.84182	12.47596	24-25 October 2018
